# Unrecognized controls on microbial functioning in Blue Carbon ecosystems: The role of mineral enzyme stabilization and allochthonous substrate supply

**DOI:** 10.1002/ece3.5962

**Published:** 2020-01-07

**Authors:** Peter Mueller, Dirk Granse, Stefanie Nolte, Magdalena Weingartner, Stefan Hoth, Kai Jensen

**Affiliations:** ^1^ Institute of Soil Science Universität Hamburg Hamburg Germany; ^2^ Applied Plant Ecology Institute of Plant Science and Microbiology Universität Hamburg Hamburg Germany; ^3^ School of Environmental Sciences University of East Anglia Norwich United Kingdom; ^4^ Centre for Environment, Fisheries and Aquaculture Science Lowestoft United Kingdom; ^5^ Molecular Plant Physiology, Institute of Plant Science and Microbiology Universität Hamburg Hamburg Germany

**Keywords:** carbon sequestration, exo‐enzymes, fungi, quantitative PCR, Indicator of Reduction in Soils, salt marsh, sea‐level rise

## Abstract

Tidal wetlands are effective carbon sinks, mitigating climate change through the long‐term removal of atmospheric CO_2_. Studies along surface‐elevation and thus flooding‐frequency gradients in tidal wetlands are often used to understand the effects of accelerated sea‐level rise on carbon sequestration, a process that is primarily determined by the balance of primary production and microbial decomposition. It has often been hypothesized that rates of microbial decomposition would increase with elevation and associated increases in soil oxygen availability; however, previous studies yield a wide range of outcomes and equivocal results. Our mechanistic understanding of the elevation–decomposition relationship is limited because most effort has been devoted to understanding the terminal steps of the decomposition process. A few studies assessed microbial exo‐enzyme activities (EEAs) as initial and rate‐limiting steps that often reveal important insight into microbial energy and nutrient constraints. The present study assessed EEAs and microbial abundance along a coastal ecotone stretching a flooding gradient from tidal flat to high marsh in the European Wadden Sea. We found that stabilization of exo‐enzymes to mineral sediments leads to high specific EEAs at low substrate concentrations in frequently flooded, sediment‐rich zones of the studied ecotone. We argue that the high background activity of a mineral‐associated enzyme pool provides a stable decomposition matrix in highly dynamic, frequently flooded zones. Furthermore, we demonstrate that microbial communities are less nutrient limited in frequently flooded zones, where inputs of nutrient‐rich marine organic matter are higher. This was reflected in both increasing exo‐enzymatic carbon versus nutrient acquisition and decreasing fungal versus bacterial abundance with increasing flooding frequency. Our findings thereby suggest two previously unrecognized mechanisms that may contribute to stimulated microbial activity despite decreasing oxygen availability in response to accelerated sea‐level rise.

## Graphical abstract

The present study assessed exo‐enyzme activities (EEAs) and microbial abundance along a coastal ecotone stretching a flooding gradient from tidal flat to high marsh. We found that stabilization of exo‐enzymes to mineral sediments leads to high specific EEAs at low substrate concentrations in frequently flooded, sediment‐rich zones of the studied ecotone. Furthermore, we demonstrate that microbial communities are less nutrient limited in frequently flooded zones, where inputs of nutrient‐rich marine organic matter are higher. This was reflected in both increasing exo‐enzymatic carbon versus nutrient acquisition and decreasing fungal versus bacterial abundance with increasing flooding frequency.

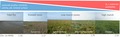

## 1 INTRODUCTION

Tidal wetlands, such as marshes and mangroves, and other vegetated coastal ecosystems have been increasingly recognized as hotspots for carbon (C) sequestration, mitigating climate change through the long‐term removal of atmospheric CO_2_ (Chmura, Anisfeld, Cahoon, & Lynch, 2003; Mcleod et al., 2011). These so‐called *Blue Carbon* ecosystems occupy <0.5% of the ocean's surface, but account for ~50% of the total C sequestration in marine sediments (Duarte, 2017; Duarte, Losada, Hendriks, Mazarrasa, & Marba, 2013). C sequestration in tidal wetlands depends on the balance of organic matter input and output, processes that are primarily controlled by net primary production (NPP) and microbial decomposition, respectively (Chmura et al., 2003; Mcleod et al., 2011). However, the important ecosystem service of C sequestration is expected to be strongly influenced by several global‐change factors, such as accelerated rates of sea‐level rise (Kirwan & Megonigal, 2013; Osland et al., 2016; Watanabe, Seike, Kajihara, Montani, & Kuwae, 2019).

Surface elevation is a master variable in coastal wetland ecology, controlling hydrology and the exchange of energy, matter, and nutrients between the wetland and the adjacent marine system, which often results in a clear plant‐species zonation along the elevation gradient (Bockelmann, Bakker, Neuhaus, & Lage, 2002). Studies along elevation gradients provide important insight into the ecosystem response to changing sea level. For instance, clear and reproducible patterns of NPP have been described in relation to surface elevation that are also represented in century‐scale forecast models on ecosystem stability and C sequestration (Kirwan & Guntenspergen, 2012; Langley, Mozdzer, Shepard, Hagerty, & Megonigal, 2013; Morris, Sundareshwar, Nietch, Kjerfve, & Cahoon, 2002; Redelstein, Dinter, Hertel, & Leuschner, 2018). By contrast, studies on microbial decomposition have produced a wide range of outcomes and thereby introduce high uncertainty to existing models (Kirwan, Langley, Guntenspergen, & Megonigal, 2013; Mueller, Jensen, & Megonigal, 2016).

It has often been hypothesized that rates of microbial decomposition would increase with elevation and associated increases in soil oxygen availability (Kirwan et al., 2013; Miller, Neubauer, & Anderson, 2001; Reed, 1995). However, the elevation–decomposition relationship is more complex because surface elevation does not only affect soil oxygen availability via flooding frequency, but also the nutrient status of the soil system by controlling the supply of dissolved inorganic nutrients and nutrient‐rich marine organic matter (Mueller et al., 2017, 2018). Moreover, organic/mineral matter contents, ion supply via salt‐water influx, and soil pH often co‐vary with surface elevation and are known to affect microbial functioning (Morrissey, Gillespie, Morina, & Franklin, 2014; Sinsabaugh et al., 2008; Weintraub, Wieder, Cleveland, & Townsend, 2013), thus potentially counterbalancing effects of oxygen availability on decomposition along elevation gradients.

Our mechanistic understanding of the elevation–decomposition relationship in tidal wetlands is limited because the majority of decomposition studies assesses CO_2_ (and CH_4_) production or the mass loss of organic matter with time and thereby integrates over the entire decomposition process by quantifying the net flux of organic C or matter (e.g., Janousek et al., 2017; Kirwan et al., 2013; Sutton‐Grier, Keller, Koch, Gilmour, & Megonigal, 2011). By comparison, insights into factors controlling the initial steps of decomposition in tidal wetlands, for example, microbial exo‐enzyme activity (EEA), are scarce (Kirwan & Megonigal, 2013; but see Morrissey, Berrier, Neubauer, & Franklin, 2013, Keuskamp, Feller, Laanbroek, Verhoeven, & Hefting, 2015). The breakdown of organic macromolecules into smaller units by microbial exo‐enzymes is considered the rate‐limiting step of the decomposition process (Sinsabaugh, Antibus, & Linkins, 1991; Sinsabaugh, Hill, & Follstad Shah, 2009). Because exo‐enzymes are substrate specific, their activity is not only a measure for the rate at which decomposition can proceed; it also represents the microbial demand for different nutrients, thus revealing insight into the energy and nutrient constraints on the overall organic matter decomposition process (Arnosti et al., 2014; Sinsabaugh et al., 2008, 2009).

Models linking EEAs to decomposition show that activities of enzymes involved in the breakdown of related substrate groups are highly correlated, so that often single indicator enzymes are used to understand microbial C and nutrient acquisition (Moorhead, Rinkes, Sinsabaugh, & Weintraub, 2013). For instance, the most frequently assayed exo‐enzymes in both marine and terrestrial systems are ß‐glucosidase, which is considered as the key enzyme in microbial C acquisition (breakdown of ß‐linked polysaccharides), leucine aminopeptidase, representing the breakdown of proteins and thus microbial N acquisition, and acid (alkaline) phosphatase, mediating microbial phosphate acquisition from various substrates (Arnosti et al., 2014). Quantifying the breakdown of different substrate types separately could be particularly relevant to understand decomposition processes in coastal ecosystems. Here, relatively nutrient‐poor and C‐rich autochthonous organic matter (vascular plant produced) and nutrient‐rich, C‐poor allochthonous organic matter (marine‐derived) contribute to different amounts to the total soil or sediment organic matter pool, shaping microbial community structure and functioning (Fagervold et al., 2014; Mueller et al., 2017; Watanabe & Kuwae, 2015).

The present study aims at improving our mechanistic understanding of how surface elevation affects organic matter decomposition processes in tidal wetlands by investigating microbial EEAs and microbial abundance along a coastal ecotone stretching an elevational gradient from tidal flat to high marsh. We assayed enzymes involved in both C and nutrient (N+P) cycling in order to relate EEAs to potential differences in nutrient supply and substrate quality (marine vs. terrestrial origin). Considering functional differences between the major decomposer groups fungi and bacteria, that is, fungi having a higher C‐use efficiency and lower nutrient demand (Strickland & Rousk, 2010), we quantified the abundance of both groups separately assaying specific gene abundance.

We hypothesize (1) that EEAs will decrease with increasing surface elevation because potential positive effects of higher oxygen availability will be counterbalanced by potential negative effects of decreasing mineral matter contents, nutrient availability, salinity, and soil pH. We hypothesize (2) that the relative microbial investment in C versus N and P acquisition will decrease with elevation because nutrient availability will decrease with elevation. We expect that decreasing nutrient availability with elevation will affect the microbial structure. Specifically, we hypothesize (3) that the abundance of fungi per unit organic matter will increase, whereas the abundance of bacteria will decrease with elevation.

## 2 MATERIALS AND METHODS

### 2.1 Study site

**Figure 1:**
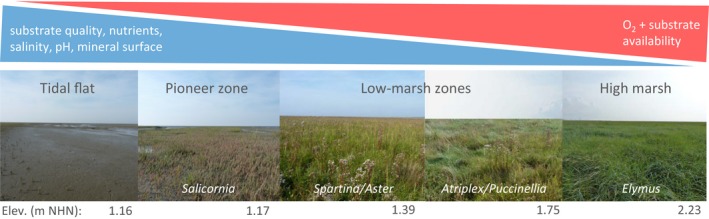
Conceptual diagram of opposing soil environmental gradients along a tidal‐flat/salt‐marsh ecotone. Photographs show sampling positions and typical plant communities along the zonation of a naturally developed salt marsh at Dieksanderkoog, German North Sea. NHN = Normalhöhennull (German standard ordnance datum) (photographs: P. Mueller)

The study was conducted in five zones along a surface‐elevation (relative sea level) gradient in a tidal‐flat/salt‐marsh ecotone on the German North Sea coast (Figure 1), situated at the outer mouth of the Elbe estuary (53°58′44.9″N 8°52′08.0″E). Zones were classified depending on plant‐species composition as (1) unvegetated tidal flat, (2) *Salicornia*‐dominated pioneer zone, (3) *Spartina*‐ and *Aster*‐dominated low marsh, (4) *Puccinellia*‐ and *Atriplex*‐dominated low marsh, and (5) *Elymus*‐dominated high marsh. The site has minerogenic sediments/soils with organic matter contents <15% and is exposed to a tidal amplitude of ca. 3.0 m.

### 2.2 Sampling and processing

Soil sampling took place in Sep 2016. From each of the five zones, eight soil samples (*n* = 8) were taken as cores from the top 5 cm using a volumetric steel ring (100 cm^3^). Sampling points within zones were randomly selected and were separated by a distance of 10–20 m. Until further analysis, intact soil cores were kept frozen at −20°C within 8 hr after sampling. A 20‐g subsample of each core was homogenized by suspending and mixing it in 20 ml deionized, ultrapure water. The resulting slurry was stored at −20°C and used as base material for further analysis (DNA extraction and PCR, element and isotope analyses, EEA assays). The residual sample was air‐dried at 65°C for 48 hr and used to determine dry mass, bulk density, organic/mineral matter content, pH, and salinity (see below).

### 2.3 Characterization of soil environmental parameters

To characterize the oxidation–reduction state and thus oxygen availability of the five sampled zones, soil redox measurements were conducted during three measuring campaigns within 6 weeks after soil sampling. Redox was measured at 5 cm depth in three randomly selected points per zone (*n* = 3) using a Pt‐tipped redox electrode and an Ag/AgCl reference electrode (ecoTech). Data were corrected to the potential of the standard hydrogen electrode to yield Eh (+207 mV). Eh data were not corrected for pH because a linear Eh‐pH relationship in soils is questionable (Mansfeldt, 2003). Soil Eh is highly variable, for example, with changing hydrological conditions. To assess this variability, we measured Eh twice after normal high‐water events and once after spring tide. Additionally, we integrated redox conditions over the 6‐week period using the Indicator of Reduction in Soils (IRIS) technique following Castenson and Rabenhorst (2006) and Rabenhorst (2008) with slight modifications (Figure S1).

Soil pH was determined in CaCl_2_ solution (10 g of air‐dried soil incubated in 25 ml of 10 mmol/L CaCl_2_). Soil salinity was approximated from electrical conductivity measured in 1:5 (air‐dried soil:DI water) dilutions following (Corwin & Yemoto, 2017). Organic/mineral matter contents of samples were determined by loss on ignition (LOI). Approximately 15 g of predried soil (105°C to constant mass) was ignited at 550°C for 2.5 hr and cooled in a desiccator (Wang, Li, & Wang, 2017). C and N contents as well as δ^13^C of the organic fraction were determined to assess microbial substrate quality and source. C, N, and δ^13^C were determined on an element analyzer (EURO‐EA 3000, Euro Vector) coupled to an isotope‐ratio mass spectrometer (Nu Horizon, Nu Instruments). δ^13^C was determined on acidified (10% HCl) samples to remove carbonates. Total phosphorus (P) of the samples was determined using an inductively coupled plasma optical‐emission spectrometer (ICP‐OES; SPECTRO ARCOS).

### 2.4 Exo‐enzyme assays

Potential EEA of ß‐glucosidase (GLU), leucine aminopeptidase (PEP), chitinase (CHI), and phosphatase (PHO) was determined in fluorometric assays following Mueller et al. (2017). GLU is considered the key enzyme of microbial C acquisition (breakdown of ß‐linked polysaccharides) in both marine and terrestrial ecosystems (Arnosti et al., 2014). PEP and CHI are considered key enzymes in the microbial acquisition of N, and PHO is the key enzyme in microbial P acquisition (Arnosti et al., 2014; Moorhead et al., 2013). We used the ratio of GLU/PEP+CHI and GLU/PHO activity to assess the relative microbial C versus nutrient demand (sensu Sinsabaugh et al., 2008, 2009). EEAs were measured close to environmental pH in 50 mmol/L bicarbonate buffer at pH 8 (Sinsabaugh et al., 2003). Unless otherwise stated, activity rates refer to normalized EEAs per unit organic matter (specific EEA), not absolute values per unit dry weight, to obtain a measure for organic matter decomposition rate (Morrissey et al., 2013, 2014).

In contrast to other studies focused on exo‐enzyme dynamics in wetland soils, we did not assess the activity of phenol oxidase (and related oxidative enzymes), which is suppressed under anoxic conditions, leading to the inhibition of other enzymes by phenolic compounds (Freeman, Ostle, & Kang, 2001). We did not assess phenol oxidase for two reasons. First, our study design would not allow separating mechanisms explaining its activity pattern because potentially lower activity in more frequently flooded zones could be caused by either inhibition through oxygen limitation or by low contents of lignin‐derived, phenol‐rich compounds in marine organic matter as opposed to terrestrial organic matter (Arnosti et al., 2014; Baldock, Masiello, Gélinas, & Hedges, 2004). Second, phenol‐rich compounds are not considered as primary C sources for any major group of microorganisms and have therefore not been included in enzyme‐acquisition ratios (Sinsabaugh et al., 2008).

### 2.5 DNA extraction and quantitative PCR

Microbial abundance was quantified assaying specific gene abundance of fungi and bacteria using qPCR following protocols outlined in Mueller et al. (2017). Briefly, DNA was extracted from a volume of soil slurry that corresponded to approximately 0.25 g dry soil, using the PowerSoil DNA extraction kit (Qiagen) following the manufacturer's protocol. SYBR Green (qPCR) assays were conducted on two technical replicates using a real‐time PCR cycler (Rotor‐Gene Q, Qiagen). Cycle threshold was automatically determined by the Rotor‐Gene Q software, and the number of gene copies was calculated using plasmid standard curves. For bacteria, the primer pair B341F/B805R was used to target the prokaryotic 16S rRNA gene region (Herlemann et al., 2011). For fungi, the primers FR1/FF390 (Chemidlin Prévost‐Bouré et al., 2011) were used targeting the fungi‐specific 18S rRNA gene region. For plasmid standard curves, DNA from cultures of *Agrobacterium tumefaciens* and *Fusarium oxysporum* were used.

### 2.6 Data analyses

As the first step of our data analyses, we conducted one‐way ANOVAs to test whether the expected gradients in microbial and soil environmental parameters existed along the five zones from tidal flat to high marsh. Kruskal–Wallis tests were used instead when ANOVA assumptions were violated (true for soil redox). The high intercorrelation of soil parameters with surface elevation in coastal ecotones does not allow for precise evaluations of the contributions of single parameters to potential differences in microbial parameters in nonmanipulative, observational studies. Therefore, in the second step of our analyses, this study focused at identifying the primary predictor for observed microbial responses using a combination of direct and partial correlation analyses following Morrissey et al. (2014) and specified below. Sampling points of the C4‐plant dominated zone 3 have been excluded from all correlation analyses including δ^13^C. Linear and nonlinear regressions were used to illustrate significant relationships. Analyses were conducted using STATISTICA 13 (Dell Software, Inc.).

To test hypothesis 1, stating that EEAs decrease with increasing elevation from tidal flat to high marsh despite increasing soil redox potentials, one‐way ANOVAs were conducted to test whether EEAs decrease and redox potentials increase along the five zones. We then identified other potential predictors of EEA responses testing for differences in pH, C:N, C:P, organic/mineral matter content, and δ^13^C along the five zones (one‐way ANOVAs) and included variables that were significantly affected by zone as predictors for EEAs in direct and partial correlation analyses. Specifically, we used direct (Pearson) correlations to identify the predictor with the strongest significant correlation to EEAs (based on *r* values). Subsequently, we tested if the correlation of the strongest predictor with EEAs remains significant when controlling for the effects of other significant predictors in partial correlations. Conversely, we also tested if other predictors remain significantly correlated when controlling for the strongest predictor.

To test hypothesis 2, stating that the relative microbial investment in C versus N and P acquisition decreases with decreasing nutrient availability from tidal flat to high marsh, one‐way ANOVAs were conducted to test for differences in EEA ratios and nutrient‐related parameters (C:N, C:P, δ^13^C) along the five zones. Identification of the primary predictor for observed responses was conducted using a combination of direct and partial correlation analyses as outlined above for hypothesis 1.

To test hypothesis 3, stating that the fungal abundance increase and bacterial abundance decrease with elevation as nutrient availability decreases, one‐way ANOVAs were conducted to test for differences in fungal abundance, bacterial abundance, and nutrient‐related parameters from tidal flat to high marsh. Direct and partial correlations were used to test for relationships between microbial abundance and nutrient availability as outlined above for hypotheses 1 and 2.

## 3 RESULTS

### 3.1 Soil environmental parameters

Eh differed significantly by zone (*p* < .0001) and was consistently lower in the tidal flat (zone 1) than in the high‐marsh zone (5), but did not consistently increase from zone 1–5 (Table 1; Figure S1). Depending on the hydrological situation (i.e., after normal tide vs. spring tide), Eh was found to be lowest in either zone 1 or the low‐marsh zone 3. Eh in zone 2 was consistently high and often higher than in zones 3–4 (Table 1; Figure S1). IRIS data (Fe(III) removal) integrating redox conditions over a 6‐week period agree well with the Eh data obtained at the three single measuring events (Figure S1). That is, Fe(III) removal, as indicator of reduction, was highest in tidal flat and low marsh and significantly lower in pioneer zone and high marsh (*p* < .05). Mean Eh of the three measuring campaigns shows a strong negative correlation with Fe(III) removal (*R* ^2^ = .852; *p* < .05), indicating that the three Eh measuring campaigns were sufficient to capture the relative differences in redox conditions between zones.

**Table 1:** Overview table of soil parameters quantified in five zones of a surface‐elevation gradient in a tidal‐flat/salt‐marsh ecotone (compare Figure 1)

Zone	(1)	(2)	(3)	(4)	(5)	*p*
OM (% mass)	3.26 ± 0.40	4.70 ± 0.18	5.08 ± 0.29	7.80 ± 0.29	11.63 ± 0.63	**.000**
δ^13^C (‰ VPDB)	−23.36 ± 0.07	−23.01 ± 0.10	−23.17 ± 0.23	−25.87 ± 0.10	−26.69 ± 0.07	**.000**
C:N (mass)	8.12 ± 0.26	8.17 ± 0.17	9.86 ± 0.23	10.30 ± 0.22	10.85 ± 0.15	**.000**
C:P (mass)	155 ± 23	158 ± 9	192 ± 16	232 ± 18	443 ± 23	**.000**
pH	7.80 ± 0.02	7.80 ± 0.02	7.75 ± 0.02	7.77 ± 0.03	7.80 ± 0.04	.546
Salinity (ppt)	33.6 ± 1.7	35.2 ± 1.0	30.5 ± 2.0	27.8 ± 2.2	21.4 ± 4.4	**.004**
Eh_normal (mV)	204 ± 56	301 ± 63	244 ± 125	300 ± 109	386 ± 30	**.000**
Eh_spring (mV)	67 ± 37	173 ± 3	−68 ± 13	33 ± 6	314 ± 11	**.000**

Note

Shown are mean values ± *SE* and *p*‐values for comparisons of mean values between zones. Pearson's *r*, significant correlations are bold‐typed at *p* ≤ .05.

Abbreviations: Eh_normal, soil redox measured after normal tide; Eh_spring, soil redox measured after spring tide; OM, organic matter.

Organic/mineral content differed significantly by zone (*p* < .0001; Table 1). Specifically, organic matter contents consistently increased by >300% from tidal flat (3.26 ± 0.40% *SE*) to high marsh (11.63 ± 0.63%). C:N strictly increased along the five zones (*p* < .0001), showing lowest values in the tidal flat (8.12 ± 0.26) and highest in the high marsh (10.85 ± 0.15) (Table 1). Similarly, C:P increased along the five zones (*p* < .0001) from 155 ± 23 in the tidal flat to 443 ± 23 in the high marsh (Table 1). δ^13^C, as indicator of the organic matter origin, decreased along the five zones (*p* < .0001), showing highest values in the tidal flat (−23.36 ± 0.07) and lowest in the high marsh (−26.69 ± 0.07) (Table 1). Regression analysis indicated that soil N was almost exclusively organically bound (Figure S2). The strongest predictor of C:N was δ^13^C (Figure S3), indicating that increases in C:N along the five zones from tidal flat to high marsh are primarily driven by decreasing relative contributions of marine organic matter to the total organic matter pool. Soil P was mainly organically bound, but our data cannot exclude that mineral contributions were also relevant (Figure S2). Likewise, the importance of marine organic input in controlling soil C:P was less clear compared to what we report for C:N (Figure S3). pH was unaffected by zone (*p* = .6; Table 1). Mean pH was 7.79 ± 0.01. Salinity significantly decreased along the five zones, showing highest values in the tidal flat and lowest in the high marsh (*p* < .0001; Table 1).

### 3.2 Microbial exo‐enzyme activities

**Figure 2:**
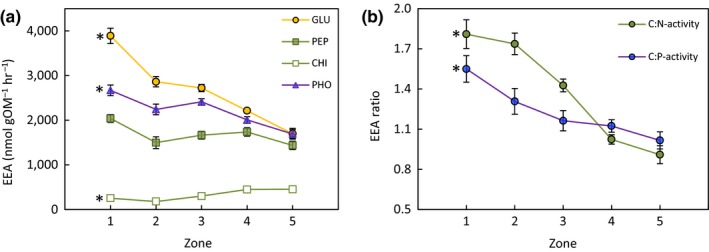
(a) Exo‐enzyme activity (EEA) of ß‐glucosidase (GLU), aminopeptidase (PEP), chitinase (CHI), and phosphatase (PHO) and (b) EEA activity ratios (C:N activity = GLU/PEP+CHI; C:P activity = GLU/PHO) along five zones (1–5) stretching an elevation gradient from tidal flat (1) to high marsh (5) (compare Figure 1). Shown are mean values ± SE. EEAs and EEA ratios that were significantly affected by zone are marked with an asterisk at *p* ≤ .05 based on 1‐way ANOVA

Specific activities of GLU and PHO strongly decreased from zone 1 to 5 (*p* < .0001; Figure 2a). Also, PEP activity tended to be lower in high marsh versus tidal flat; however, the overall effect of zone on PEP was only marginally significant (*p* = .09; Figure 2a). Only CHI activity increased from zone 1 to 5 (*p* < .0001); however, CHI activity was one order of magnitude lower than all other EEAs assayed (Figure 2a).

Nutrient‐related parameters (soil C:N, C:P, and δ^13^C) and organic/mineral matter contents were significantly correlated with the two enzymes that decreased strongest along the elevation gradient (GLU and PHO) and may thus explain the observed effect of zone on EEA. Salinity was not significantly related with GLU and PHO activity (Table 2). The high intercorrelation of parameters related with nutrient availability and organic/mineral matter content (correlations not shown) does not allow for a precise evaluation of the importance of single parameters inducing the observed decrease in EEAs along the elevation gradient. Direct and partial correlation analyses suggest organic/mineral matter content as the most influential factor for GLU and PHO activity: The strongest predictor for GLU and PHO activity (based on direct correlation analyses) was organic/mineral matter content (Table 2). When controlling for the effect of organic/mineral matter content on these EEAs in partial correlations, nutrient‐related parameters were not significantly related with GLU and PHO activity, whereas organic/mineral matter content remained significantly related when controlling for the effect of any of the other parameters (Table 2).

**Table 2:** Direct and partial correlation matrices for EEAs that significantly decreased along the five zones from tidal flat to high marsh (GLU and PHO; compare Figure 2a)

	OM	Salinity	δ^13^C	C:N	C:P
(a) Direct correlations					
GLU	**−0.838**	0.304	**0.681**	**−0.794**	**−0.723**
PHO	**−0.712**	0.121	**0.528**	**−0.593**	**−0.607**
(b) Partial correlations: OM versus EEAs controlling for factors in columns					
Corr. (OM vs. GLU)	–	**−0.790**	**−0.693**	**−0.578**	**−0.589**
Corr. (OM vs. PHO)	–	**−0.735**	**−0.622**	**−0.585**	**−0.483**
(c) Partial correlations: factors in columns versus EEAs controlling for OM					
GLU	–	−0.116	−0.249	−0.259	0.126
PHO	–	−0.293	−0.321	0.058	0.106

Note

(a) Direct correlations between specific EEAs and soil factors potentially explaining the EEA responses along the surface‐elevation gradient. (b) Correlations between EEAs and organic matter (as the strongest predictor identified in a) while controlling for other factors. (c) Correlations between EEAs and factors potentially explaining the EEA responses along the surface‐elevation gradient while controlling for the effect of organic matter (as the strongest predictor identified in a). Values represent Pearson's *r*, significant correlations are bold‐typed at *p* ≤ .05.

Abbreviation: OM, organic matter.

**Figure 3:**
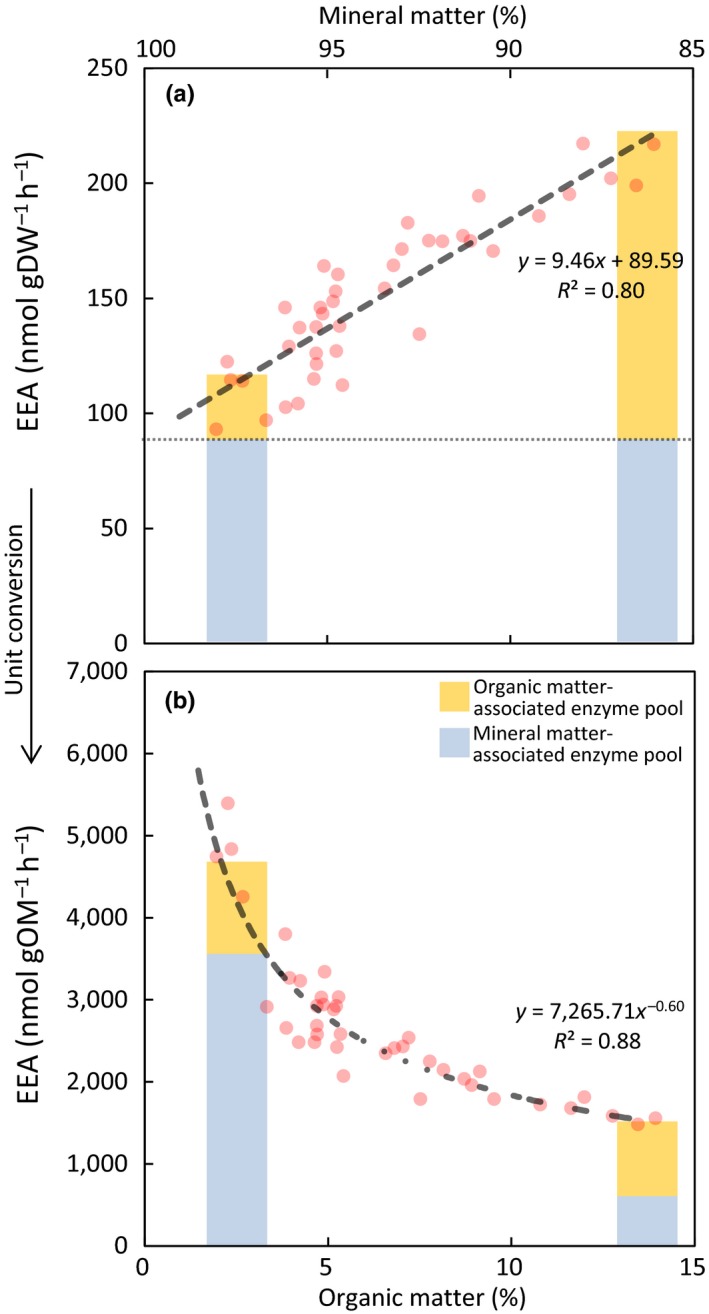
(a) Absolute exo‐enzyme activity (EEA per unit dry weight) and (b) specific activity (EEA per unit organic matter) of ß‐glucosidase versus mineral/organic matter content. Bars represent data interpretation based on the linear function *y*‐intercept in panel a, indicating a relatively constant background activity of ca. 90 nmol gDW^−1^ hr^−1^ (i.e., when organic matter approaches 0%) associated with a mineral‐bound enzyme pool. The high *y*‐intercept of the linear function in panel (a)  translates to a negative power function in panel (b) upon unit conversion from absolute to specific EEA. Bars have been converted proportionally from panel a to b. See Figure S4 for further specific EEA versus organic matter relationships

The strong negative relationships between organic matter content and specific EEAs (i.e., EEA per unit organic matter) result from high vertical (*y*‐axis) intercepts when plotting absolute enzyme activities (i.e., EEA per unit dry weight) against organic matter content (Figures 3a and S4), indicating high EEAs when organic matter contents approach zero. The high vertical intercepts of these significant and positive linear functions translate into highly correlated negative power functions between EEA and organic matter content when transforming absolute to normalized EEAs of GLU and PHO per unit organic matter (Figures 3b and S4).

### 3.3 Microbial carbon versus nutrient acquisition

Both microbial C:N activity and C:P activity decreased strongly and consistently along the elevation gradient (Figure 2b; *p* < .0001 and *p* = .03, respectively), showing highest values in the tidal flat (1.81 ± 0.11 and 1.55 ± 0.10, respectively) and lowest in the high marsh (0.91 ± 0.07 and 1.02 ± 0.06, respectively). Organic/mineral matter content, nutrient‐related parameters (C:N, C:P, δ^13^C), and salinity were significantly correlated with both C:N activity (Table 3a) and C:P activity (Table 3b).

**Table 3:** Direct and partial correlation matrices

	Direct	Partial correlations	
(a) C:N activity		Corr. ( δ^13^C vs. C:N activity)	Controlling for δ^13^C
OM	**−0.739**	**0.378**	−0.182
Salinity	**0.443**	**0.708**	0.027
δ^13^C	**0.774**	–	–
C:N	**−0.731**	**0.419**	−0.209
C:P	**−0.667**	**0.542**	−0.155
(b) C:P activity		Corr. (C:N vs. C:P activity)	Controlling for C:N
OM	**−0.631**	**−0.322**	−0.103
Salinity	**0.398**	**−0.500**	0.088
δ^13^C	**0.596**	**−0.429**	0.018
C:N	**−0.689**	–	–
C:P	**−0.566**	**−0.399**	−0.022

Note

(a) Left column: direct correlations between microbial C:N activity and soil factors potentially explaining the EEA ratio response along the surface‐elevation gradient; middle column: correlations between C:N activity and δ^13^C (as the strongest predictor identified in direct correlations) controlling for other factors (in rows); right column: correlations between C:N activity and factors potentially explaining the EEA ratio response along the surface‐elevation gradient while controlling for δ^13^C (as the strongest predictor identified in direct correlations). (b) Left column: direct correlations between microbial C:P activity and factors potentially explaining the EEA ratio response along the surface‐elevation gradient; middle column: correlations between C:P activity and soil C:N ratio (as the strongest predictor identified in direct correlations) controlling for other factors (in rows); right column: correlations between C:P activity and factors potentially explaining the EEA ratio response along the surface‐elevation gradient while controlling for soil C:N (as the strongest predictor identified in direct correlations). Values represent Pearson's *r*, bold‐typed at *p* ≤ .05.

Abbreviation: OM, organic matter.

The high intercorrelation of these parameters does not allow for a precise evaluation of the importance of single parameters inducing the observed decrease in C versus nutrient acquisition ratios along the elevation gradient. However, direct and partial correlation analyses suggest δ^13^C and C:N as the most influential factors (Table 3): The strongest predictor (based on direct correlation analyses) for C:N activity was δ^13^C (Table 3a). When controlling for the effect of δ^13^C on C:N activity in partial correlations, none of the other parameters remained significantly related, whereas δ^13^C remained significantly related when controlling for the effect of any of the other parameters (Table 3a). The strongest predictor for C:P activity was C:N (Table 3b). When controlling for the effect of C:N on C:P activity in partial correlations, none of the other parameters remained significantly related, whereas C:N remained significantly related when controlling for the effect of any of the other parameters (Table 3b).

### 3.4 Microbial abundance

**Figure 4:**
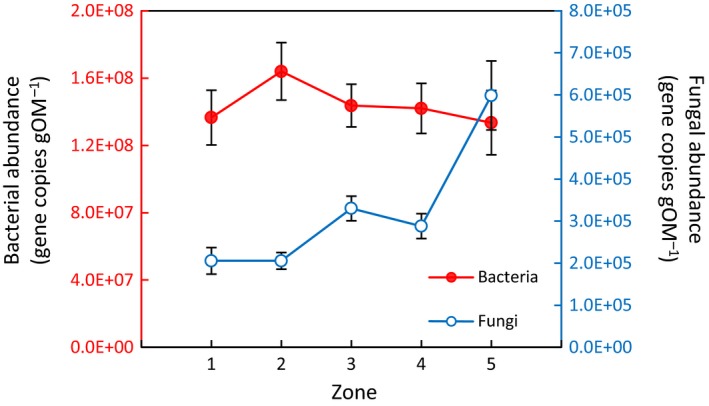
Bacterial and fungal gene abundance along five zones (1–5) stretching an elevation gradient from tidal flat (1) to high marsh (5) (compare Figure 1). Shown are mean values ± *SE*. One‐way ANOVA indicates a significant effect of zone on fungal abundance (*p* < .0001, but no effect on bacterial abundance (*p* = .755). Note separate *y*‐axes for bacterial and fungal abundance

Bacterial abundance was unaffected by zone (*p* = .8), whereas fungal abundance increased significantly along the elevational gradient (*p* < .0001), showing lowest values in tidal flat and highest in the high marsh (Figure 4). Across zones, fungal abundance was three orders of magnitude lower than bacterial abundance (Figure 4). No correlations between bacterial abundance and any of the soil parameters assessed were found (data not shown). By contrast, fungal abundance showed negative correlations with nutrient‐related parameters and salinity. C:P was the strongest predictor for fungal abundance and remained negatively related when controlling for the effects of other significant predictors (Table 4). Conversely, none of the other predictors remained significantly related to fungal abundance when controlling for C:P (Table 4).

**Table 4:** (a) Direct correlations between fungal gene abundance (per unit organic matter) and soil factors potentially explaining the fungal response along the studied elevation gradient. (b and c) Partial correlations evaluating the importance of soil C:P ratio [as the strongest predictor identified in (a)] and fungal abundance

OM	salinity	δ^13^C	C:N	C:P
(a) Factors in columns versus fungal abundance				
**0.622**	**−0.410**	**−0.591**	**0.582**	**0.712**
(b) C:P versus fungal abundance controlling for factors in columns				
**0.352**	**0.582**	**0.496**	**0.446**	–
(c) Factors in columns versus fungal abundance controlling for C:P				
−0.057	−0.060	−0.078	0.017	–

Note

Values represent Pearson's *r*, significant correlations are bold‐typed at *p* ≤ .05.

Abbreviation: OM, organic matter.

## 4 DISCUSSION

### 4.1 Specific exo‐enzyme activities decrease with elevation

In accordance with our first hypothesis, specific enzyme activities (i.e., per unit organic matter) decreased along the elevation gradient, despite clearly lower redox potentials in tidal flat and low marsh compared to the high‐marsh zone (Figure 2a). Only CHI activity diverged from this pattern, which, however, was one order of magnitude lower than all other EEAs assayed and therefore played a negligible role for microbial C and N acquisition in all zones of the studied ecotone (Figure 2a). The strongest EEA declines with elevation were found for GLU and PHO, which declined from tidal flat to high marsh by 56 and 37%, respectively.

Our findings suggest that organic matter content or its inverse—mineral matter content—is the strongest predictor for both specific GLU and PHO activities along the studied elevation gradient (Table 2, Figures 3 and S4). Although negative relationships between organic matter content and specific EEAs in tidal wetlands have previously been shown (Morrissey et al., 2014), these relationships have been interpreted differently. That is, lower organic matter contents have been interpreted as the result of higher EEAs, not vice versa, because EEA—as the rate‐limiting step of the decomposition process—was expected to control soil organic matter contents. However, it needs to be doubted for the present study that decomposition controls organic/mineral contents (i.e., >87% explained variance in case of GLU; Figure 3b) exclusively, considering the several‐fold decrease in mineral matter deposition along the elevation gradient of the studied site (Butzeck et al., 2015).

We argue that enzyme stabilization to mineral surfaces provides explanation for the observed tight relationships between specific EEAs and organic/mineral matter contents (Ensminger & Gieseking, 1942; Tietjen & Wetzel, 2003): The functions of absolute GLU and PHO activity (i.e., per unit dry weight) versus organic matter content (Figures 3 and S4) show high vertical intercepts (i.e., high EEAs when organic matter contents approach 0%) and thus provide evidence of a large mineral matter‐associated enzyme pool (Allison, 2006). When transforming absolute EEAs (per unit dry weight) to specific EEAs (per unit organic matter), the high vertical intercepts of the linear absolute‐EEA functions translate into the highly correlated negative power functions of specific EEAs versus organic matter content (Figures 3 and S4). As such, the shape of the specific EEA functions simply reflects the addition of a mineral‐associated enzyme pool to an organic matter‐associated pool (Figure 3). This mineral‐associated enzyme stabilization is mostly restricted to smaller grain sizes, that is, silts and clays (Zimmerman & Ahn, 2011). In our site, silt and clay contents represent approx. 80% of the deposited mineral matter and their contributions do not vary across the elevation gradient within the marsh (Butzeck et al., 2015; Mueller et al., 2019). In accordance with this, slopes and *y*‐intercepts of the relationships between EEA per unit dry weight and organic/mineral content are relatively consistent across zones, indicating similarly active mineral‐associated enzyme pools (Figure 3a).

Enzyme stabilization to mineral surfaces can change the kinetic properties of enzymes and often leads to a reduced catalytic activity (Allison, 2006; Zimmerman & Ahn, 2011). Our data are insufficient to support this notion. However, on a mass basis, the mineral‐associated enzyme pool shows lower activity than the organic‐associated pool. That is, at maximum GLU activity per unit dry weight (220 nmol gDW^−1^ hr^−1^), only 40% of the total activity is associated with the mineral fraction, which represents 86% of the total soil mass, and 60% of the activity is associated with the organic fraction, corresponding to only 14% of the total soil mass (Figure 3a). In addition to affecting enzyme kinetic properties, stabilization to mineral surfaces can considerably increase enzyme life spans, which has been proposed as a strategy to acquire energy and nutrients in soil systems characterized by a high variability of substrate availability in space and time (Weintraub et al., 2013; Zoppini & Marxsen, 2011). This may be particularly true for intertidal ecosystems, where tide‐induced changes in soil moisture, substrate supply, and O_2_ availability induce a high spatiotemporal variability in microbial substrate accessibility and utilization (D'Andrea, Aller, & Lopez, 2002; McClain et al., 2003; Werner et al., 2006). We therefore argue that enzyme stabilization to minerals could provide a stable decomposition matrix in tidal‐wetland soils, which enables a rapid microbial utilization of substrates during short phases of frequently re‐occurring suitable environmental conditions.

**Figure 5:**
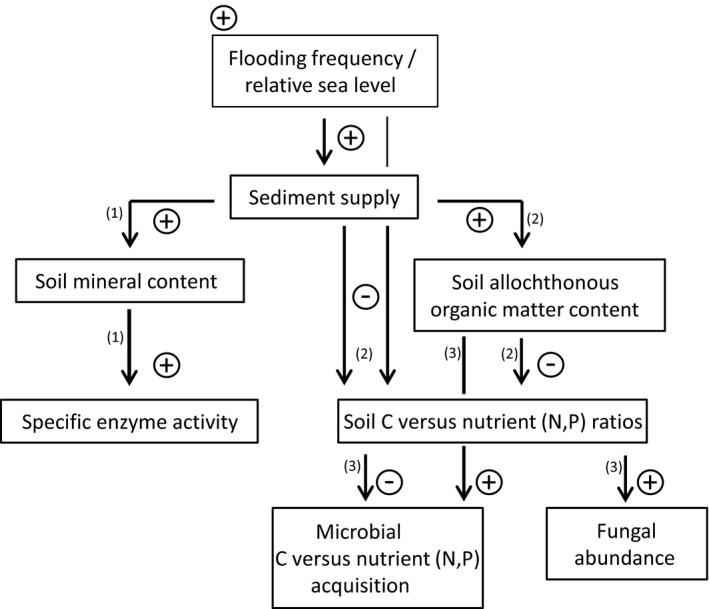
Conceptual diagram of flooding‐frequency effects on microbial structure and exo‐enzyme activity (EEA) in a minerogenic salt‐marsh ecosystem. ± symbols on arrows indicate positive/negative relationships between parameters in boxes as exemplified in the following: (1) Our data provide evidence of a mineral matter‐associated enzyme pool leading to high specific EEAs at low organic matter contents (Table 2; Figures 3 and S4). We argue that enzyme stabilization to minerals could enable rapid microbial utilization of substrates during short phases of frequently re‐occurring suitable environmental conditions in tidal wetlands. (2) Higher flooding frequency increases inorganic and organic nutrient (N+P) supply to the soil system. Soil N is almost exclusively organically bound and strongly controlled by the supply of N‐rich allochthonous organic matter (Figures S2 and S3). The importance of allochthonous organic input for P supply is less clear (Figures S2 and S3). (3) Nutrient availability and allochthonous organic input determine microbial C versus nutrient acquisition and specific fungal abundance (Tables 3 and 4)

Soil clay content is widely used as a predictor for C storage in terrestrial biogeochemical models, because clay promotes the sorption of organic matter to mineral surfaces and aggregate formation (Rasmussen et al., 2018). However, the relevance of this mechanism for C sequestration in tidal wetlands has been questioned (Kirwan & Megonigal, 2013). Even in mineral‐dominated NW European salt marshes, sedimentary clay inputs do not seem to enhance the stabilization of autochthonous organic matter (Spohn, Babka, & Giani, 2013; Van de Broek et al., 2018). Instead, the present study provides evidence of a previously unrecognized function of sedimentary mineral inputs in tidal wetlands by illustrating remarkably tight correlations between organic/mineral matter content and EEAs (Figure 5).

### 4.2 Microbial carbon versus nutrient acquisition

The investigation of EEAs to understand decomposition processes in soils can be particularly insightful because EEAs associated with the acquisition of C and nutrients can be put in relation and reveal important insight into the microbial energy versus nutrient demand (Sinsabaugh et al., 2008, 2009). In accordance with our second hypothesis, the relative microbial investment in C versus N and P acquisition decreased strongly with deceasing nutrient availability along the studied elevation gradient from tidal flat to high marsh, indicating an increasingly greater nutrient versus C demand of the microbial community with elevation (Figure 2b). Importantly, our findings further suggest that this nutrient gradient controlling microbial functioning was primarily driven by the decreasing contribution of marine‐derived (allochthonous) organic matter with increasing elevation. This is illustrated by several lines of evidence, including highly significant direct and partial correlations between organic matter δ^13^C and microbial C:N activity (Table 3), soil C:N ratios and microbial C:P activity (Table 3), and δ^13^C and soil C:N ratios (Figure S3).

Due to drastically diverging compositions of organic matter, relationships of C and nutrient acquisition are expected to markedly differ between the terrestrial and the marine environment (Arnosti et al., 2014). Marine organic matter is characterized by a far lower proportion of C‐rich structural components and shows lower C:N and C:P ratios than terrestrial organic matter (Baldock et al., 2004; Cleveland & Liptzin, 2007; Khan, Vane, & Horton, 2015; McGroddy, Daufresne, & Hedin, 2004). Because tidal wetlands are situated at the interface of the terrestrial and the marine environment, pronounced gradients in microbial C versus nutrient acquisition can be expected along transects that bridge the two environments and are clearly supported by our data (Figure 2b). Although the relevance of marine organic matter as microbial C source in tidal wetlands has long been demonstrated (Boschker, Brouwer, & Cappenberg, 1999), its effects on biogeochemical processes in tidal‐wetland soils are poorly understood (Bouillon et al., 2004; Mueller et al., 2017; Van de Broek et al., 2018). Our data suggest that microbial functioning in the topsoil of the studied minerogenic tidal‐wetland system is strongly controlled by the mixing of allochthonous and autochthonous organic matter in response to differences in flooding frequency. We therefore argue that the influence of allochthonous organic matter supply on microbial element cycling in tidal wetlands requires more attention, particularly with regard to accelerated rates of sea‐level rise (Figure 5).

### 4.3 Fungal abundance increases with elevation

In partial accordance with our third hypothesis, we demonstrate an increasing specific soil fungal abundance along the studied elevation gradient from tidal flat to high marsh (Figure 4), whereas bacterial abundance remained constant along this gradient and in relation to all here assessed environmental parameters. The abundance of fungal decomposer communities in terrestrial soils can have important implications for nutrient and energy fluxes and is an important component of terrestrial food‐web models and concepts (Moore et al., 2004; Strickland & Rousk, 2010). However, whether findings from terrestrial systems concerning the ecological functions of fungal decomposer communities are applicable to tidal‐wetland soils is unclear, because the majority of studies from wetlands and tidal wetlands focused on fungi associated with standing dead biomass or surface litter (Hyde & Lee, 1995; da Luz Calado & Margarida, 2012; Yarwood, 2018).

In terrestrial soils, fungi are often associated with a higher C‐use efficiency and a lower nutrient demand than bacteria (van der Heijden, Bardgett, & Straalen, 2008; Strickland & Rousk, 2010), and thus, nutrient cycles are supposed to become increasingly closed (i.e., less leaky) and C turnover to slow down with increasing relative fungal abundance (Martínez‐García, Deyn, Pugnaire, Kothamasi, & van der Heijden, 2017; Moore et al., 2004; de Vries, Hoffland, Eekeren, Brussaard, & Bloem, 2006; Wardle, Bardgett, et al., 2004; Wardle, Walker, & Bardgett, 2004). The here presented increase in fungal abundance along the elevation gradient might therefore reflect that nutrient cycles in tidal wetlands become increasingly closed with decreasing flooding frequency and thus external nutrient input (Schrama, Jouta, Berg, & Olff, 2013; Van Wijnen & Bakker, 2000). In support of this concept, direct and partial correlations indeed suggest that nutrient‐related parameters—and particularly soil C:P—exert primary control on fungal abundance along the studied elevation gradient (Table 4).

Other factors previously discussed to affect the fungal abundance in tidal‐wetland soils are salinity and oxygen availability (Bossio & Scow, 1998; Chambers, Guevara, Boyer, Troxler, & Davis, 2016; Hyde & Lee, 1995). Although our data suggest a weakly negative correlation of salinity and fungal abundance, this relationship does not remain significant when controlling for other environmental parameters co‐varying along the elevation gradient (Table 4). Unfortunately, our sampling design did not allow to test for correlations between oxygen availability (or redox conditions) and fungal abundance. However, we do not see differences in fungal abundance between tidal flat and pioneer zone despite consistent and large differences in soil redox conditions among the zones (Table 1, Figure S1). Additionally, we previously demonstrated that fungal abundance is unresponsive to regularly occurring, large differences in soil redox (ΔEh > 100 mV) in higher elevated areas of the studied site (Mueller et al., 2017). We therefore conclude that decreasing marine‐derived nutrient input, rather than decreasing soil salinity or increasing oxygen availability, is the primary driver of increasing fungal abundance with elevation in the studied system (Figure 5).

The primers used for our qPCR assays do not allow separating saprotrophic from symbiotic fungal (i.e., arbuscular mycorrhizae) communities, which makes it difficult to interpret links between microbial structure and functioning. Arbuscular mycorrhizae are known to support wetland‐plant growth particularly through their provision of phosphate (Yarwood, 2018), and the importance of their interaction with plants has also been demonstrated for tidal wetlands (Burke, Hamerlynck, & Hahn, 2003; Daleo et al., 2007). It is therefore possible that the strong correlation between soil C:P ratios and fungal abundance reflects an increase in arbuscular mycorrhizae abundance along the elevation gradient.

### 4.4 Sea‐level rise implications

A growing number of studies assessing the impact of accelerated sea‐level rise on tidal‐wetland C cycling demonstrate either negligible effects or even stimulated microbial decomposition in response to increasing flooding frequency (Mueller et al., 2018 and refs. therein). The mechanisms driving such unexpected results are currently not well understood (Kirwan et al., 2013). The present work suggests at least two previously unrecognized mechanisms that may contribute to stimulated microbial activity in response to increasing flooding frequency. First, we demonstrate that the stabilization of exo‐enzymes to mineral sediments leads to high specific EEAs at low substrate concentrations in frequently flooded, sediment‐rich zones of the studied ecotone. The high background activity of a mineral‐associated enzyme pool could provide a stable decomposition matrix in these highly dynamic zones, which enables a rapid microbial utilization of substrates during short phases of frequently re‐occurring suitable environmental conditions. Second, we demonstrate that microbial communities are less nutrient limited in frequently flooded zones, because inputs of nutrient‐rich marine organic matter (and possibly inorganic nutrients) are higher. This is reflected in both increasing exo‐enzymatic C versus nutrient acquisition and decreasing fungal versus bacterial abundance with increasing flooding frequency. In order to integrate these findings into our current mechanistic understanding of tidal‐wetland C cycling, future research will need to assess how changes in microbial EEA and EEA ratios relate to the final steps of the mineralization process (i.e., CO_2_ production) (Billings & Ballantyne, 2013; Morrissey et al., 2013) and if an increasing fungal abundance in soils is related to slower C turnover rates, as suggested for terrestrial ecosystems (van der Heijden et al., 2008; Martínez‐García et al., 2017; Strickland & Rousk, 2010).

## CONFLICT OF INTEREST

None declared.

## AUTHOR CONTRIBUTIONS

PM, SN, SH, and KJ designed the study. PM conducted field and laboratory work. DG and MW planned and conducted molecular work. PM analyzed all data and wrote the manuscript with comments provided by all other co‐authors.

## Data Availability

The data used in this work are available at the DRYAD data repository https://doi.org/10.5061/dryad.p5hqbzkm1.
