# Supplementary material for: Unrecognized controls on microbial functioning in Blue Carbon ecosystems: The role of mineral enzyme stabilization and allochthonous substrate supply
Source: Ecol Evol. 2020 Jan 7;10(2):998–1011. doi: 10.1002/ece3.5962 (PMC6988540; doi:10.1002/ece3.5962)
Supplement: Supplementary file 1 [file ECE3-10-998-s001.pdf]

Figure S1

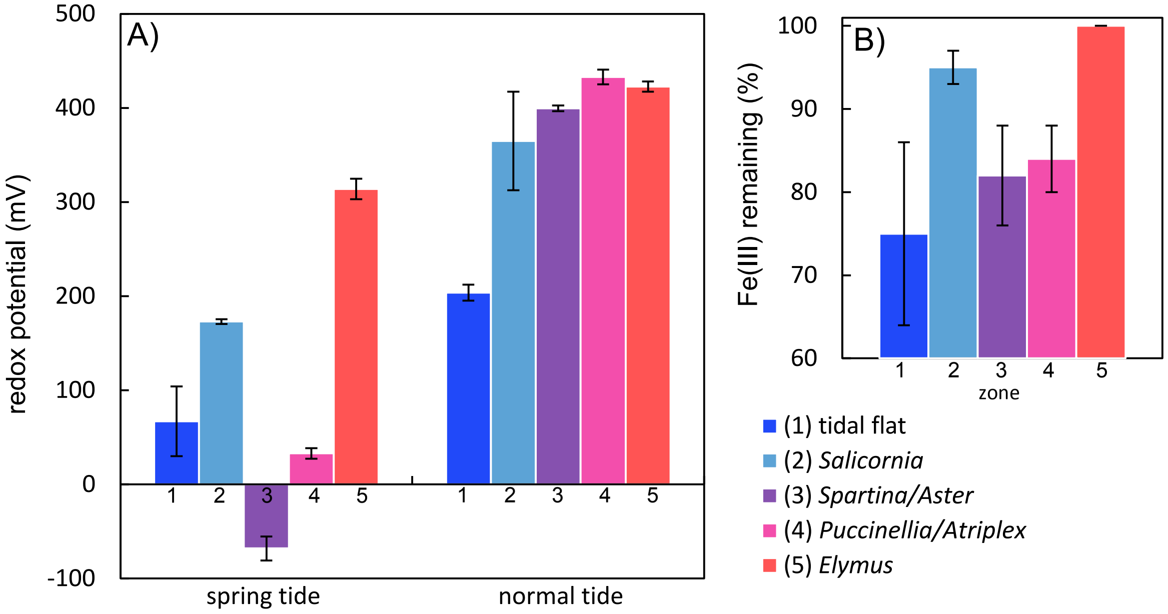

Supplementary Figure S1 [**A**] Soil redox at 5 cm depth measured during low water after normal high tide (mean of two measuring campaigns) and after spring tide (one measuring campaign). [**B**] Percentage Fe(III) remaining on IRIS sticks to a soil depth of 20 cm after a deployment phase of 6 weeks. Presented are mean values ± standard errors (n = 3). IRIS-method summary: Finely sanded PVC pieces of 5 x 40 cm were coated with Fe(III) paint and inserted to a depth of 25 cm. As water saturated soils become progressively reduced, microbes oxidizing organic matter will reduce solid phase Fe(III) (iron oxides) to soluble Fe(II). This will dissolve and consequently remove the iron oxide paint applied to the PVC surface, indicating reducing soil conditions (Rabenhorst 2008). After field incubation, IRIS sticks were gently cleaned, and IMG-file format images of the sticks were obtained using an image scanner. Paint removal was quantified using the open source software Fiji (ImageJ, GNU).

Figure S2

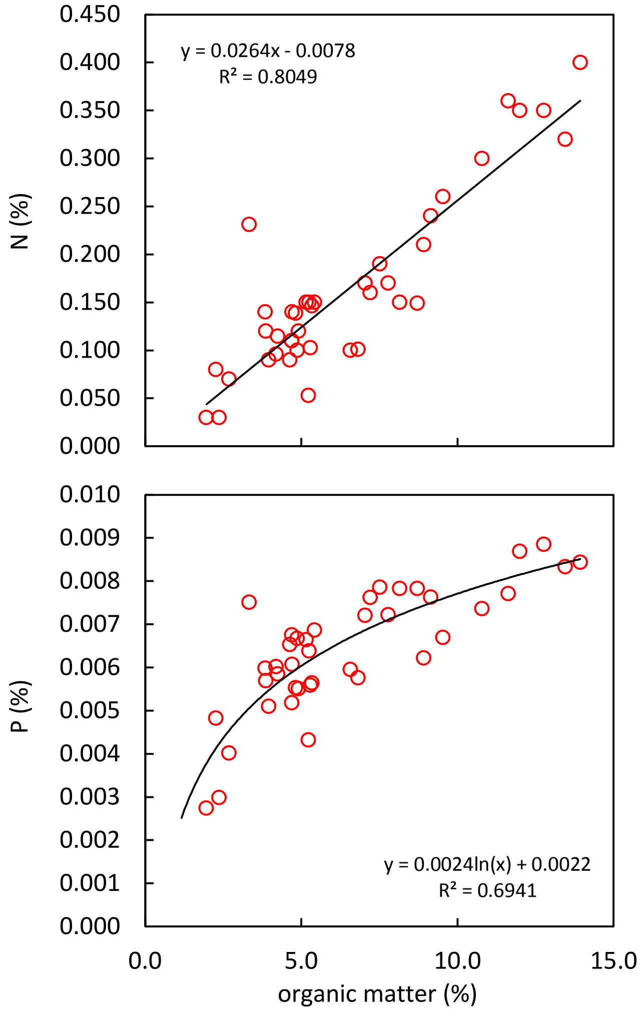

Supplementary Figure S2 Soil total nitrogen (N) and phosphorous (P) contents in relation to organic matter contents.

Figure S3

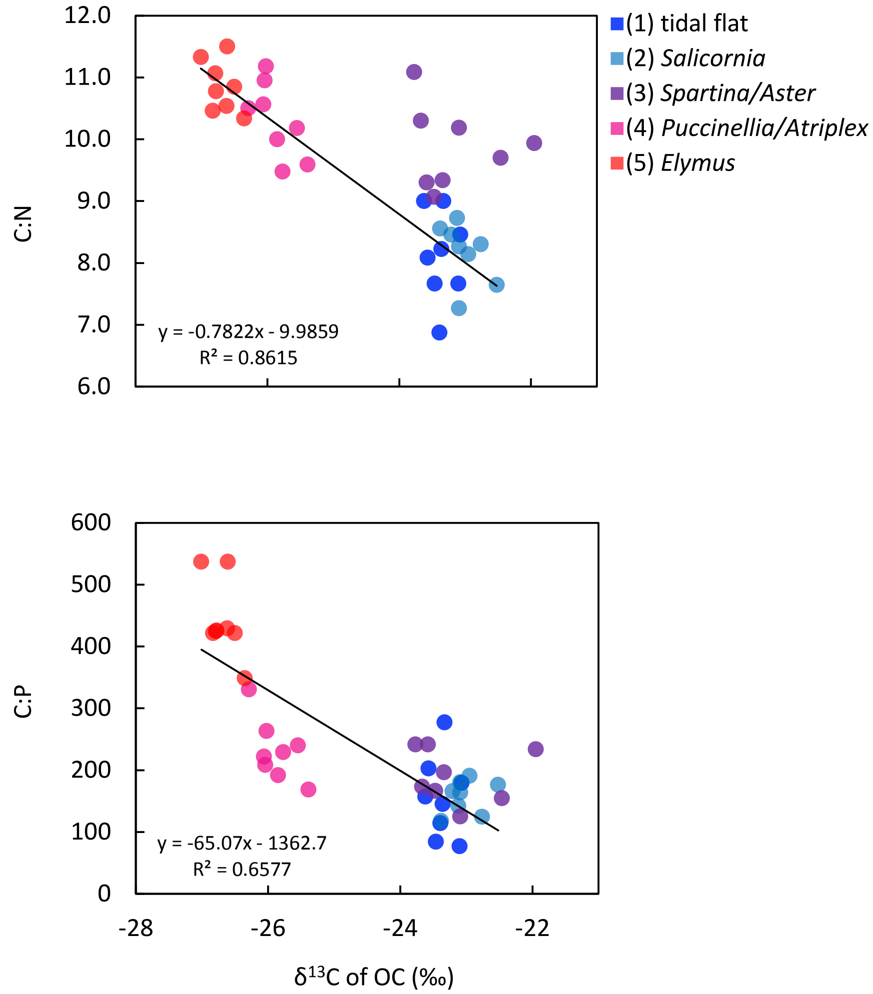

Supplementary Figure S3 Soil C:N and C:P ratios in relation to the δ13C signature of organic carbon. Data points of the C4-grass dominated zone 3 have been excluded from regression analyses.

Figure S4

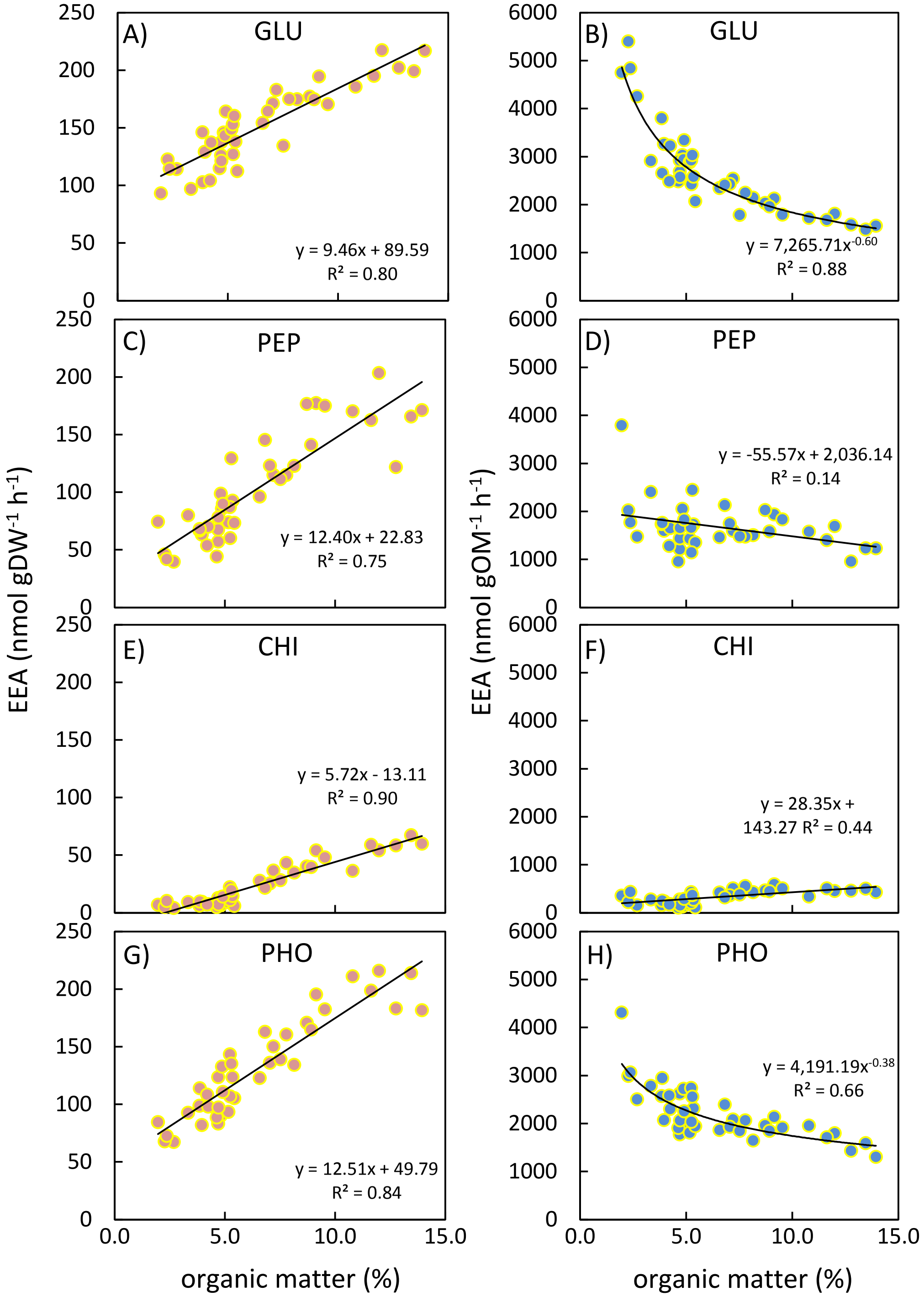

Supplementary Figure S4 Absolute (left panels) and specific (right panels) exo-enzyme activitis of ß-glucosidase (GLU, [A+B]), peptidase (PEP, [C+D]), chitinase (CHI, [E+F]), and phosphatase (PHO, [G+H]) vs. organic matter content.
